# The role of dendritic cells and their immunometabolism in rheumatoid arthritis

**DOI:** 10.3389/fimmu.2023.1161148

**Published:** 2023-05-12

**Authors:** Yuichi Suwa, Yasuo Nagafuchi, Saeko Yamada, Keishi Fujio

**Affiliations:** ^1^ Department of Allergy and Rheumatology, Graduate School of Medicine, The University of Tokyo, Tokyo, Japan; ^2^ Department of Functional Genomics and Immunological Diseases, Graduate School of Medicine, The University of Tokyo, Tokyo, Japan

**Keywords:** rheumatoid arthritis, immunometabolism, dendritic cell (DC), glycolysis (glycolytic pathway), OXPHOS (oxidative phosphorylation)

## Abstract

Dendritic cells (DCs) play crucial roles in the pathogenesis of rheumatoid arthritis (RA), a prototypic autoimmune disease characterized by chronic synovitis and joint destruction. Conventional dendritic cells (cDCs) with professional antigen-presenting functions are enriched in the RA synovium. In the synovium, the cDCs are activated and show both enhanced migratory capacities and T cell activation in comparison with peripheral blood cDCs. Plasmacytoid dendritic cells, another subtype of DCs capable of type I interferon production, are likely to be tolerogenic in RA. Monocyte-derived dendritic cells (moDCs), once called “inflammatory DCs”, are localized in the RA synovium, and they induce T-helper 17 cell expansion and enhanced proinflammatory cytokine production. Recent studies revealed that synovial proinflammatory hypoxic environments are linked to metabolic reprogramming. Activation of cDCs in the RA synovium is accompanied by enhanced glycolysis and anabolism. In sharp contrast, promoting catabolism can induce tolerogenic DCs from monocytes. Herein, we review recent studies that address the roles of DCs and their immunometabolic features in RA. Immunometabolism of DCs could be a potential therapeutic target in RA.

## Introduction

Rheumatoid arthritis (RA) is a chronic autoimmune disease characterized by systemic synovitis and sometimes accompanied by progressive bone destruction (1). Joint deformities lead to a loss of mobility in RA patients, and treatment-related adverse events including cardiovascular events and infections can be fatal (2–5). While various anti-rheumatic drugs have been established in recent years, 5 - 20% of RA patients have poor responses to those medications (6). The pathogenesis of RA is thought to involve a reduced tolerance of the autoimmune system resulting from genetic and environmental backgrounds (7, 8). Synovitis in RA is induced by complex interaction of various cell types, including T and B lymphocytes involved in adaptive immunity, myeloid cells involved mainly in innate immunity, osteoclasts and synovial fibroblasts directly responsible for joint destruction. The complexity of disease pathogenesis is a primary cause of the difficulties in treatment (9–11). Dendritic cells (DCs), a subtype of the myeloid lineage, could be related to the clinical treatment response in RA. Recently, we have reported that the proportion of precursor DCs (pre-DCs) in RA peripheral blood has a strong association with treatment resistance, and their gene signature is a better predictor of response than established clinical parameters such as anti-citrullinated peptide autoantibodies (ACPA) and duration of disease (12). Understanding DCs is essential for elucidation of RA pathogenesis and developing new treatment strategies.

## The role of dendritic cells in rheumatoid arthritis

**Figure 1:**
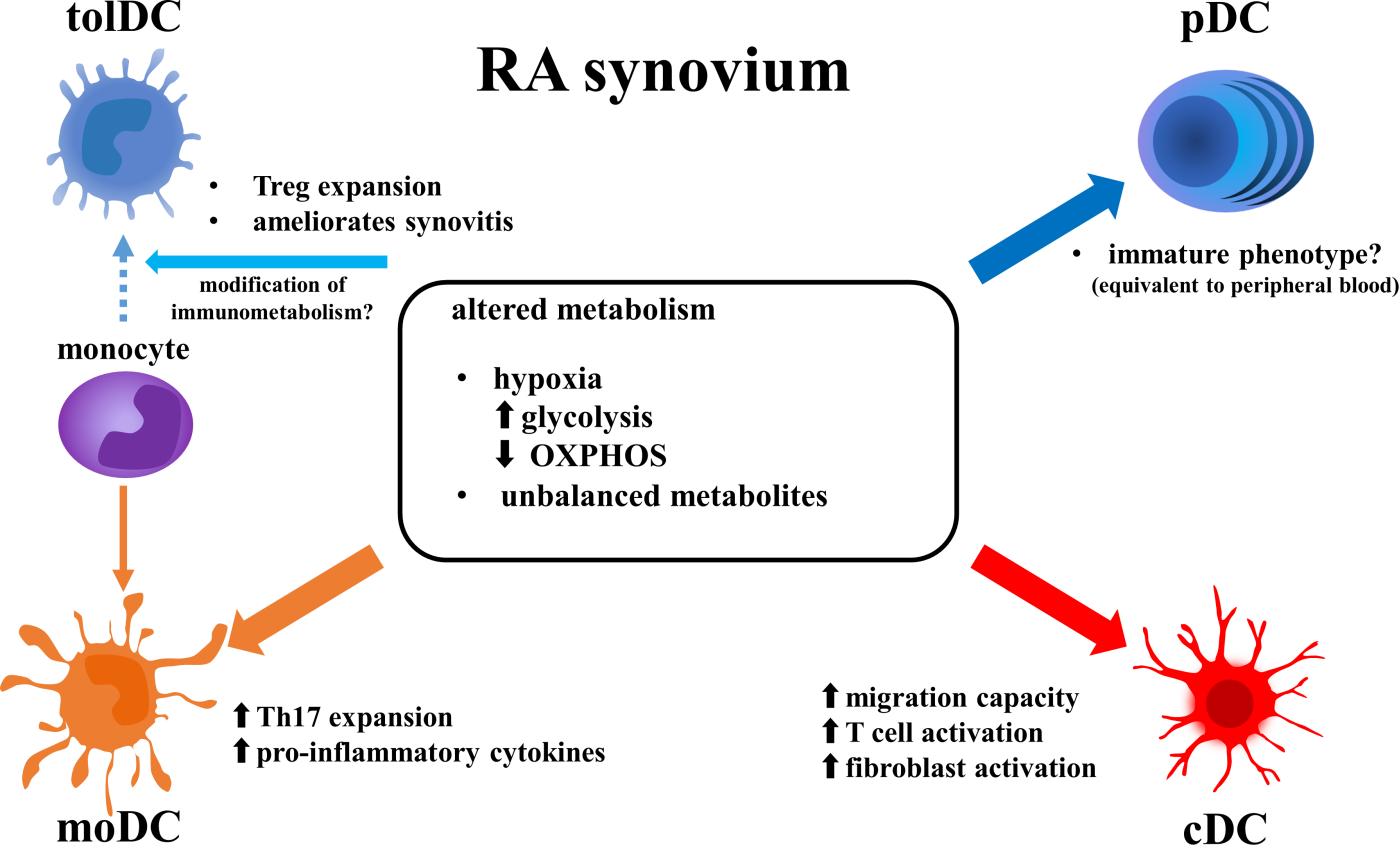
Altered function of DC subsets in RA synovium. Metabolic features in RA synovium alter phenotype of DC subsets. Enhanced glycolysis and reduced OXPHOS induce pro-inflammatory function in cDCs and moDCs, while they may suppress activation of pDCs. Modification of metabolism in RA synovium may induce differentiation of monocytes to tolDCs instead of moDCs. DCs, dendritic cells; RA, rheumatoid arthritis; tolDCm, tolerogenic DC; pDC, plasmacytoid DC; moDC, monocyte-derived DC; cDC, conventional DC; OXPHOS, oxidative phosphorylation; Treg, regulatory T cell; Th17, T-helper17 cell; IFN, interferon.

DCs are cells with specialized antigen-presenting capabilities (13). Flow cytometry and single cell analysis has permitted the classification of various subsets of DCs (**Figure 1**). Conventional DCs (cDCs) bearing a specific marker (CD11c) and plasmacytoid DCs (pDCs) expressing CD123, constitute the two major subsets of human DCs. cDCs are further classified into cDC1 and cDC2. cDC1 express high levels of CD141 and possess an intrinsic capacity for cross-presentation *via* MHC class I to activate CD8^+^ T cells. cDC2 is the dominant subtype of cDC, with high expression of CD1c and a robust capacity for activating CD4^+^ T cells (14). On the other hand, the pDC subset is specialized to respond to viral infection by rapid production of high quantities of type I interferons (IFNs) and secretion of cytokines. Several reports have shown that cDCs promote joint inflammation in RA. For example, transient depletion of cDCs reduced arthritis in CD11cDTR transgenic mice (15). In human RA synovial fluid (RASF), the frequency of cDC2s is higher than in peripheral blood. They have increased expression of antigen-presenting and co-stimulatory molecules, and coculture with T cells induced the latter to proliferate and secrete IFNγ, interleukin-4 (IL-4) and IL-17 (16). On the other hand, the role of pDCs in RA is only partially understood. Several reports have suggested that pDCs act in a preventive manner, reducing inflammation. Depletion of pDCs in a Balb/c mouse model promoted arthritis (17). Single-cell RNA-sequencing (scRNA-seq) analysis of RA peripheral blood revealed a pDC cluster with an activated transcriptomic profile (high gene expression associated with TLR, IFN regulatory factors and chemokine receptors). However, its proportion was not correlated with disease activity. Instead, patients with inactive RA had higher frequencies of a pDC cluster with a “healthy transcriptomic profile” than did patients with active RA (18). In RASF, pDC has been reported to exhibit an immature phenotype. The pDC found in SF demonstrated low expression of the co-stimulation markers CD40, CD80 and CD83, comparable to that found in peripheral blood, whereas SF cDC displayed higher expression of these markers compared to peripheral blood cDC (19).

In addition to those two major subsets, several new subsets have been described. Monocyte-derived DCs (moDCs) are induced from monocytes *in vivo.* They have proinflammatory functions and are positive for monocytic marker CD14. They likely correspond to “inflammatory DC” observed in local inflamed tissue, including the RA synovium (20). Human moDCs were reported to induce naïve CD4^+^ T cells to become helper T17 cells (Th17) and cause synovitis (21). Conversely, tolerogenic DCs (tolDCs) can be generated from monocytes during exposure to growth factors, cytokines or pharmacologic agents (22). In Balb/cAnNCrl model mice, tolDCs reduced arthritis scores *via* modulation of naïve CD4^+^ T cells and induced expansion of regulatory T cells (Treg) (23).

Single-cell analysis has revealed the dynamics of DC in RA synovia. Wu et al. performed scRNA-seq analysis of ACPA+ and ACPA- RA patients before treatment, and they identified eight DC subtypes. Those subtypes in RA synovia included three cDC subtypes, two pDC subtypes, and “DC_macrophages” (a CD14 positive subset). HLA-DR5, CCL3, SPP1 and BRI3 expression was upregulated in synovial DC of ACPA+ RA. CCL13, CCL18 and MMP3 expression was upregulated in synovial DC of ACPA- RA (24).

As described above, each DC subset has a distinct function and a different phenotypic feature in RA. The mechanism by which DC functions are altered in RA is likely linked to disease pathogenesis. Previous studies showed that the metabolism of immune cells closely affects the pathogenesis of autoimmune diseases (25, 26). RA is also reported to have some metabolic features. Here, we focus on metabolic features of RA joints and their relation to DC function.

## Metabolic features of joints in rheumatoid arthritis

Altered cellular metabolism in the microenvironment of joints in RA has been described. The serum metabolite signatures differed when healthy controls (HCs) were compared to RA patients (27). In another report, the activities of metabolic enzymes differed in RA synovia. Activities of glyceraldehyde 3- phosphate dehydrogenase (GAPDH) and lactate dehydrogenase (LDH) were higher in RA synovial cells than those collected during trauma surgery. Moreover, metabolic profiles were associated with disease activity of RA. The lactate/glucose ratio of SF was higher in RA than in both ankylosing spondylitis (AS) and psoriatic arthritis (PsA) patients, and it correlated with joint symptoms (28). The metabolite signature of RASF had different features than other arthritic diseases (AS, Behçet’s disease and gout), with increased metabolites of the tricarboxylic acid (TCA) cycle and decreased lipid metabolites (29). The specific state of the microenvironment in RA joints gives rise to these metabolic features. For example, the RA synovium is exposed to elevated hypoxia (30). Consequently, there is a higher production of hypoxia inducible factor alpha (HIFα) than in HCs (31). HIFα promotes glycolysis by increasing the expression of enzymes and transporters involved in the glycolytic pathway, such as glucose transporter 1 (GLUT1) and hexokinase 2 (HK2) (32, 33). Altered metabolism affects the functions of cells present in RA joints. The metabolites of glycolytic pathways activate pro-inflammatory processes of immune cells (34), and HIFα activation promotes inflammation and bone destruction *in vitro* (35, 36).

In addition to glycolysis, several other metabolic changes associated with mitochondrial dysfunction have been reported in RA (37). The hypoxic state in RA joints results in a disturbance of the TCA cycle, with an accumulation of succinate (29). Hypoxia also induces production of reactive oxygen species (ROS) (38). RA synovial fluid had higher levels of ROS and mitochondrial DNA mutations, and the extent correlated with inflammation and the level of SF tumor necrosis factor alpha (TNFα) (39). *In vitro*, the hypoxic state elevated glycolytic enzymes and mitochondrial dysfunction in synovial cells from RA, both of which were ameliorated by TNFα inhibitors (36). Genes involved in mitochondrial fission were also upregulated in RA synovia (40). Mitochondrial fission results in enhanced glycolysis instead of mitochondrial respiration (oxidative phosphorylation [OXPHOS]) (41). Indeed, the RA synovium had lower OXPHOS than did that from HCs (42).

Compared to glucose and mitochondrial metabolism, less is known about lipid metabolism. However, several studies described lipid metabolic features of RA. In individuals who had a high risk of developing RA, the serum lipidome profile resembled that of patients with active RA (43). In transcriptomic analysis of synovial tissues of high-risk individuals, genes involved in lipid metabolism were down-regulated only in the individuals who developed RA, whereas the expression levels of genes involved in mitochondrial respirations were not altered (44). In addition, mitochondrial fatty acid oxidation (FAO), which is necessary for lipid utilization in the TCA cycle, was significantly impaired in both RA and RA-high risk synovia, with an increased dependence on glucose oxidation (45). Therefore, lipid metabolism may be involved in the pathogenesis of RA. These various metabolic features in the joints of RA patients may affect immune cell functions.

## Immunometabolism of DC is relevant to the pathogenesis of RA

Metabolism plays an important role in the activation of DCs. In general, anabolism enhances immunogenicity, whereas catabolism induces tolerogenicity (46). In cultured murine DCs, activation *via* stimulation of toll like receptors (TLRs) caused stable DCs to change their energy dependence from OXPHOS to glycolysis, which allowed them to immediately obtain the energy needed for inflammatory mediator production and their antigen-presenting capacities (47). Impaired OXPHOS and FAO and enhanced glycolysis by hypoxic RA synovia may lead to anabolism and enhance immunogenicity of synovial DCs. Several reports on cDCs and moDCs in RA synovia support that hypothesis. cDC1 in RA synovia had higher gene expression of the hypoxia marker TREM-1, and induced higher levels of T cell activation that subsequently enhanced secretion of pro-inflammatory cytokines and activated synovial fibroblasts, compared to peripheral blood cDC1 (48). With respect to cDC2, gene expression of glycolytic pathways was upregulated in cDC2 of RA synovia compared to peripheral blood cells (49). Synovial cDC2 of RA and PsA patients had enhanced migratory capacity, and had higher levels of both costimulatory and coinhibitory markers in another report (16). MoDCs, treated with supernatants from cultured RA synovia *ex vivo*, also showed upregulated glycolytic pathways with higher IL-1 and IL-12 production, and expression of adhesion and co-stimulation molecules (ICAM and CD83) (49). Furthermore, DCs also responded to metabolites and altered their immunogenicity (50, 51). Succinate, which is an intermediate metabolite of the TCA cycle, is higher in RA synovia than in other types of inflammatory arthritis (29). DCs can sense succinate *via* succinate receptor (GPR91) in synergy with TLRs to activate their inflammatory functions (52). DCs with GPR91 deficiency reduced Th17 expansion and reduced the development of arthritis in mice (53).

If the environment of RA synovia inhibits mitochondrial respiration (OXPHOS) and enhances glycolysis, these changes may explain why cDCs and moDCs have proinflammatory roles whereas pDCs have a less inflammatory role in RA joints (40). Upon activation by stimulated TLR7/8, human cDC2s and pDCs depend on different metabolic pathways as energy sources. For example, cDC2s expressed genes that are associated with mitochondrial fission and glycolysis, whereas pDCs expressed genes associated with mitochondrial fusion and OXPHOS (54). Similarly, human moDCs displayed lower gene expression levels associated with catabolic pathways including FAO and OXPHOS compared to induced tolDCs (55). These reports suggest that the metabolic features of RA joints modulate DC functions (**Figure 1**).

## Immunometabolism of DC as candidate therapeutic targets of RA

Various therapeutic agents have been developed to block chemical signals or proteins involved in the inflammatory pathway in RA, e.g., TNF inhibitors (TNFi), IL-6 receptor inhibitors (IL-6Ri), Janus kinase inhibitors (JAKi), anti-CD20 drug and co-stimulation molecules (CD80/86) inhibitor. However, the number of cases who reached disease remission with response to each agent is limited from 10% to 50% (56). Some RA patients have limited treatment choices because of adverse events and co-morbidities. The patient population in which RA does not reach low disease activity or remission even after switching treatments is called “difficult-to-treat” (D2T) RA, which is a challenge for clinical settings in rheumatology (57).

Some immunologically targeted agents for RA show a correlation between treatment response and immunometabolism. A recent study examined the characteristics of synovial biopsies and clinical responsiveness to treatment with tocilizumab (IL-6Ri) or rituximab (anti-CD20 monoclonal antibody) for TNFi-resistant RA. In tocilizumab responders, the PPARγ signaling pathway, which is involved in lipid metabolism, was enhanced together with upregulation of myeloid cell cytokine module (58). Tofacitinib (TOF), a JAKi, enhanced mitochondrial OXPHOS and suppressed glycolysis, a response consistent with a decrease in inflammatory cytokines in RA synovial explants (59). Clinical RA improvement by TOF correlated with the reduction of activated STAT-3 in RA synovium, which is known to play a role in DC maturation (49, 60). TOF reduced the T cell stimulatory capacity of human moDC *in vitro* (61). In SKG mice, a genetic model of RA, TOF induced tolDCs and ameliorated arthritis (62). Thus, metabolism plays an important role in the function of immune cells, and may become a new therapeutic target of RA.

Glycolysis is essential for activation of proinflammatory DCs. In SKG mice, inhibition of HK2, which is an initiator of glycolysis, suppressed DC activation *via* stimulation of TLR by lipopolysaccharide, inhibited T cell differentiation to Th17 with Treg expansion and ameliorated ongoing arthritis (63). Although the immunometabolism of tolDCs is not adequately understood, control of metabolism can induce tolerogenicity in DCs. Metformin, a drug for diabetes, induces DCs to enter a catabolic state (activate OXPHOS and PI3K/Akt/mTOR pathway as well as glycolysis) *via* the AMPK pathway, and promotes an anti-inflammatory process (64). The use of metformin slightly improved DAS-CRP28 in RA patients compared to placebo (65). Vitamin D also activates AMPK pathway and induces a catabolic state in DCs, promotes the formation of tolDCs (66). Deficiency of the active form of vitamin D has been reported to correlate with disease activity in systemic lupus erythematosus (SLE) (67), and its supplementation improved disease activity in SLE patients (68). TolDCs, which were derived from peripheral blood monocytes of RA patients with the use of vitamin D, suppressed activation of CD4+ T cells (69, 70). Therefore, supplementation with the active form of vitamin D may have beneficial effects on RA through induction of tolDCs.

Heterogeneous aspects of RA, such as age of onset, comorbidities, extra-articular organ involvement, seropositivity of rheumatoid factor or ACPA, limit treatment options. These disease characteristics cause populations of D2T RA, regardless of the many available therapeutic agents (57). Metabolism of RA joints has unique characteristics that differ from those of peripheral blood in RA or from synovia of other types of inflammatory arthritis (29, 49). In addition, some metabolic enzymes have an isotype whose expression is upregulated in specific immune cells (71). Regulating the immunometabolism of cell populations that are found only in inflamed RA joints may avoid excessive suppression of normal immune cells. Therefore, agents targeting immunometabolism can be disease- and organ- specific treatments.

## Conclusion

DCs play a crucial role in the pathogenesis of RA. Herein, we summarized each subset of DCs and the alteration of their functions by metabolic changes (**Figure 1**). In the RA synovium, hypoxia, enhanced glycolysis, suppression of OXPHOS and decreased lipid metabolism induce pro-inflammatory activity in DCs. The immunometabolism of DCs may represent a new target for precision medicine, since some RA patients have myeloid-dominant cell infiltrations in the synovium (58).

However, we need to be careful in the interpretation of current studies of the immunometabolism of RA. Typical studies of the metabolism of RA patients had small sample sizes. Moreover, most reports showing a link between modulation of metabolism and amelioration of arthritis have been conducted *in vitro* or in murine models of RA. There have been no therapeutic agents reported which target specifically the immunometabolism of DCs, and ameliorate arthritis. Furthermore, the immunometabolism in actual RA synovium may be more intricate than previously thought, as it is influenced by various biological pathways, metabolites and cells in the microenvironment and their effects with one another (72). In the RA synovium, each space and layer possesses resident immune cells and fibroblasts that have specific functions and different frequencies due to different tissue microenvironments (73). Increased understanding of immunometabolism in RA will be achieved by combining single cell analysis, description of the metabolome and three-dimensional special transcriptomics using human samples (74–77).

## Author contributions

YS, YN, SY, KF designed the study and contributed to writing the manuscript. KF supervised the study. All authors were involved in drafting the article or revising it critically for important intellectual content, and all authors approved the final version to be published. All authors contributed to the article and approved the submitted version.
